# Supplementary material for: Disentangling conical intersection and coherent molecular dynamics in methyl bromide with attosecond transient absorption spectroscopy
Source: Nat Commun. 2019 Jul 16;10:3133. doi: 10.1038/s41467-019-10789-7 (PMC6635414; doi:10.1038/s41467-019-10789-7)
Supplement: Supplementary file 1 — Supplementary Information [file 41467_2019_10789_MOESM1_ESM.pdf]

Online Supplementary Material for "Disentangling conical intersection and coherent molecular dynamics in methyl bromide with attosecond transient absorption spectroscopy"

Timmers *et al.*

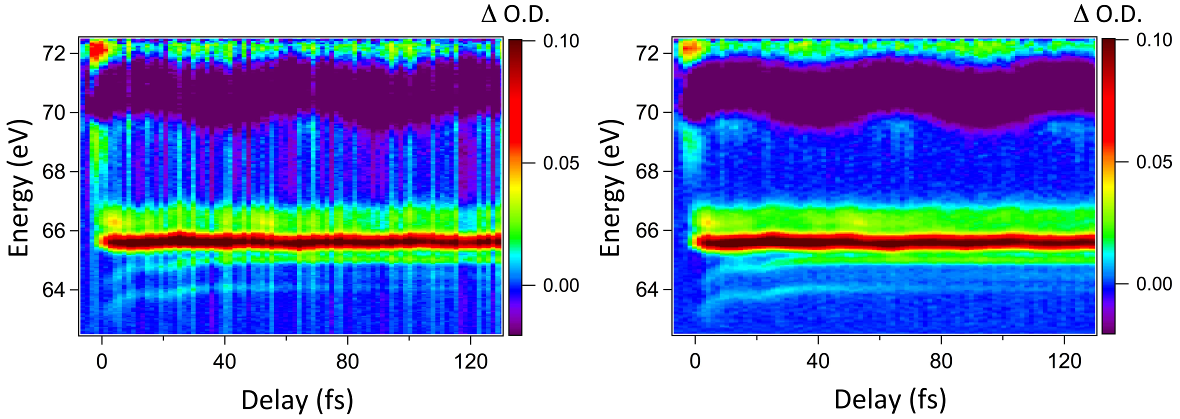

Supplementary Figure 1: Comparison of the experimental ATAS spectrogram before (left) and after (right) the linear subtraction post-processing to remove the transient absorbance noise from CEP instability.

Supplementary Note 1: Analysis of Process 1

Spin-orbit Transitions

Previous experiments investigating the $M_{4,5}$ edge of atomic Br have reported the observation of three absorption features: (i) Br ($P_{3/2} \rightarrow D_{5/2}$) (64.4 eV), (ii) Br* ($P_{1/2} \rightarrow D_{3/2}$) (65 eV), and (iii) Br ($P_{3/2} \rightarrow D_{3/2}$) (65.4 eV) [1]. In the manuscript, we also observe three atomic absorption features. However, we do not assign these transitions to the features listed above. Instead we assign the lowest energy feature to the dipole forbidden Br* ($P_{1/2} \rightarrow D_{5/2}$) (64.1 eV) and the next two transitions to lines (i) and (ii). As mentioned in the manuscript, the transition restriction in the Br* ($P_{1/2} \rightarrow D_{5/2}$) absorption feature is lifted in the molecule due to the Br atom coupling to an unpaired electron in the methyl group. This allows us to see the absorption feature for a finite time delay before the molecule completely dissociates. From simulation (Fig. 2), we also find that the Br ($P_{3/2} \rightarrow D_{3/2}$) (65.4 eV) transition exhibits the weakest transition strength of the four spin-orbit transitions. Finally, experimentally, the Br ($P_{3/2} \rightarrow D_{3/2}$) transition is totally obscured by the strongest transition line corresponding to the L1 transition in the molecular ion.

Measuring the Bifurcation Time

Due to the congestion of different neutral transitions and limited signal-to-noise, it is rather difficult to directly observe the bifurcation of the excited state wavepacket in the experimental ATAS trace between the $^3Q_{0^+}$ and $^1Q_1$ excited states compared against the simulated ATAS trace. However, the growth of Br yield into the Br ($P_{3/2} \rightarrow D_{5/2}$) transition indicates that a transition has occurred. Therefore, to extract out the bifurcation time, we must first remove the more prominent Br* transitions in order to unambiguously measure the dynamics giving rise to Br atomic yield. This is done by fitting and subtracting a bi-Gaussian curve to the Br* ($P_{1/2} \rightarrow D_{5/2}$) and ($P_{1/2} \rightarrow D_{3/2}$) transitions at each delay point in the experimental

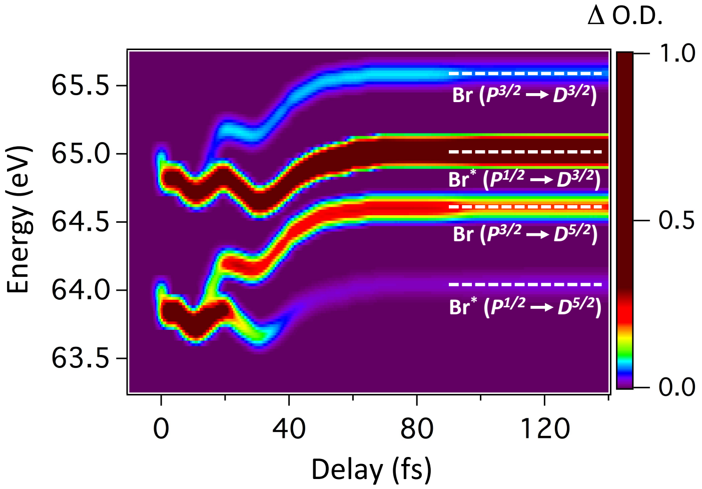

Supplementary Figure 2: Full simulated ATAS trace showing all four spin-orbit transitions in the dissociation of the neutral molecule.

ATAS trace. It should be noted that the data was first smoothed in order to properly fit and subtract the bi-Gaussian curves. The subtracted trace is shown on the left hand side of Fig. 3. With this Br*-subtracted trace, we can now sum directly over the energy range of 64-65 eV in order to measure the dynamics giving rise to Br atomic yield. This sum is shown on the right side of Fig. 3, demonstrating a clear exponential rise. The sum is fit to an exponential curve,

$$f(x) = A\left[1 - e^{-(x-x_0)/\tau}\right], \tag{1}$$

in order to extract an appearance time of $x_0 = 8 \pm 3$ fs for the bifurcation event. However, this quantity corresponds to when the Br yield emerges from 0 Δ O.D. and does not correspond with the center-of-mass bifurcation time measured from the simulated trace. To measure this quantity, we first observe that the residual for the exponential fit [inset of Fig. 3] exhibits a Gaussian anomaly shortly after the appearance time of the wavepacket. By fitting this residual to a Gaussian function, we measure a center-of-mass appearance time of $\tau_b = 15.0 \pm 0.4$ fs.

Photo-dissociation Time of the $^3Q_{0^+}$ State

Since the neutral absorption feature converging to Br* ($P_{1/2} \rightarrow D_{5/2}$) is dipole forbidden in the atomic limit due to spin-orbit selection rules, the absorption strength gives a clear, model-independent measure of the fragmentation of the $^3Q_{0^+}$ excited state of the molecule into atomic Br. Therefore, we can fit the data after 35 fs (when it has fully converged to its final energetic position) to an exponential curve in order to extract out the photo-dissociation time of the $^3Q_{0^+}$ state. Fig. 4 plots a lineout of this absorption feature along with an exponential fit corresponding to a measured dissociation time of $\tau = 68 \pm 3$ fs.

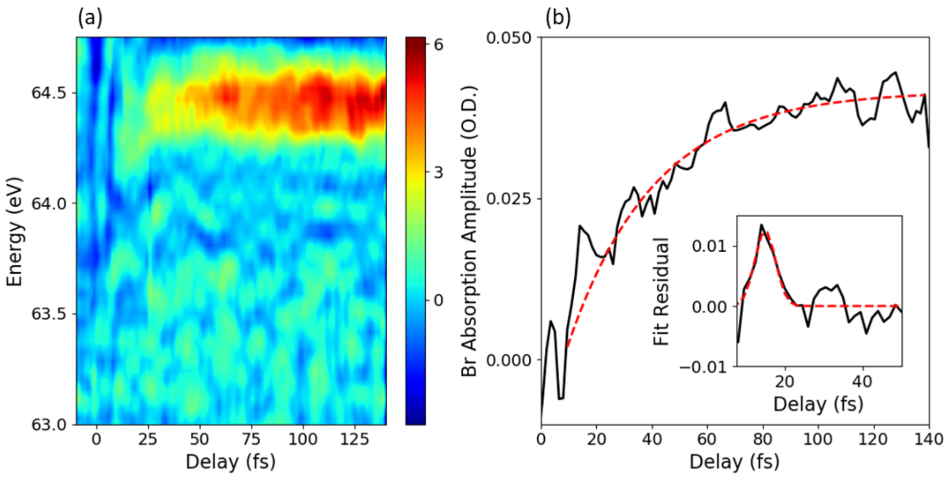

Supplementary Figure 3: (a) Experimental ATAS trace for process I in which the Br* ($P_{1/2} \rightarrow D_{5/2}$) and ($P_{1/2} \rightarrow D_{3/2}$) transitions have been subtracted out. (b) A sum over the Br ($P_{3/2} \rightarrow D_{5/2}$) curve from 64-65 eV, demonstrating the exponential rise of the Br yield. When the sum is fit to an exponential curve (red dashed), the residual (inset plot) depicts a clear Gaussian shape corresponding to a bifurcation time of $\tau_b = 15.0 \pm 0.4$ fs.

Supplementary Note 2: Mechanism for Process 2

As noted in the manuscript, the strong field creation of a ground state vibrational wavepacket accounting for process 2 can be created by a thermal (Lochfraß) or non-thermal (stimulated Raman or bond softening) strong field excitation mechanism. The thermal mechanism termed as Lochfraß is based upon the R-dependent ionization rate of a thermally excited vibrational manifold, where R is the bond coordinate for this vibrational manifold. Since higher vibrational states see an effectively lower ionization potential at higher R values, the ionization rate will be higher for these states. This will lead to the creation of a coherent hole in the thermal distribution of vibrational states. However, for Lochfraß to work, there needs to be a fairly substantial thermal population. In a previous study reporting the observation of Lochfraß excitation in Br_2, Hosler *et al.* [2] found that the excited vibrational population accounted for $> 20\%$ of the total vibrational population in Br_2 at 300 K. However, in the present experiment, the reduced mass of CH_3Br is lighter resulting in a higher vibrational frequency. Therefore, the excited vibrational population accounts for $< 5\%$ of the total vibrational population in CH_3Br at 300 K. In addition, Lochfraß predicts the creation of the wavepacket at an outer turning point on the ground state potential energy surface where the ionization rate is highest. This means the center-of-energy oscillation of the wavepacket should start at an extreme, exhibiting a cosine-like phase. However, the oscillation measured in the present experiment exhibits a sine-like phase. All these results strongly indicate Lochfraß to be an unlikely mechanism.

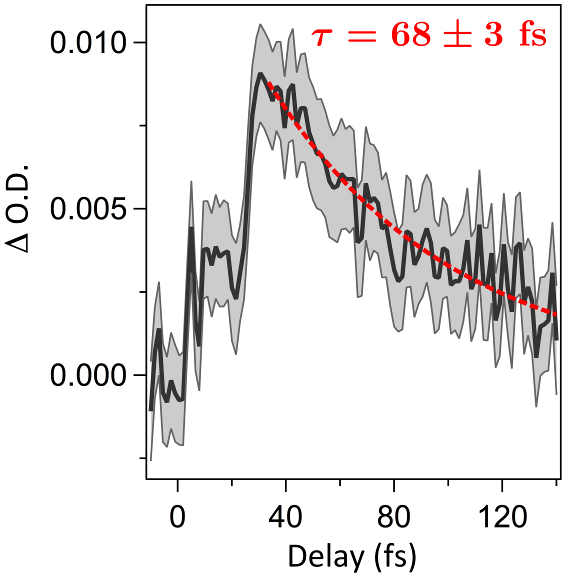

Supplementary Figure 4: Lineout of the spin-orbit forbidden absorption feature corresponding to Br* ($P^{1/2} \rightarrow D^{5/2}$). The decrease of the signal past 40 fs is a clear measure of the transition of the molecule into its atomic fragment, yielding a photo-dissociation time of $\tau = 68 \pm 3$ fs.

Supplementary Note 3: Theoretical Simulations of Attosecond Transient Absorption Dynamics

The adiabatic energies for both the excited and Rydberg states of CH_3Br were calculated using State-Averaged Complete Active Space Self-Consistent Field (SA-CASSCF) theory using Molpro[3]. The active space consisted of C-Br σ bonding and anti-bonding orbitals, p_x and p_y lone pair orbitals on Br, and the 5s Rydberg orbital. The aug-cc-pVTZ-pp basis set and the associated Relativistic Effective Core Potential(RECP) are used for Br[4], and the aug-cc-pVTZ basis set for C and H atoms. An additional diffuse function with exponential factors 0.023 was included to improve the description of 5s Rydberg states. These exponential factors were optimized to minimize the average energy of the corresponding Rydberg states. This choice of basis set, including the Rydberg orbitals, is largely consistent with previous calculations on the system[5], with the exception that higher angular momentum basis functions are fully included in this study. Spin orbit-couplings were calculated from the RECP[4]. Without spin-orbit interactions, parallel transitions from the ground electronic state to $\pi \rightarrow \sigma^*$ and $\pi \rightarrow 5s$ states are symmetry forbidden. Spin-orbit couplings cause the ground, $\pi \rightarrow \sigma^*$, and $\sigma \rightarrow \sigma^*$ states to mix, creating the $^3Q_{0+}$ state. The $^3Q_{0+}$ state is the only valence excited state with a symmetry allowed transition along the parallel direction from the ground state.

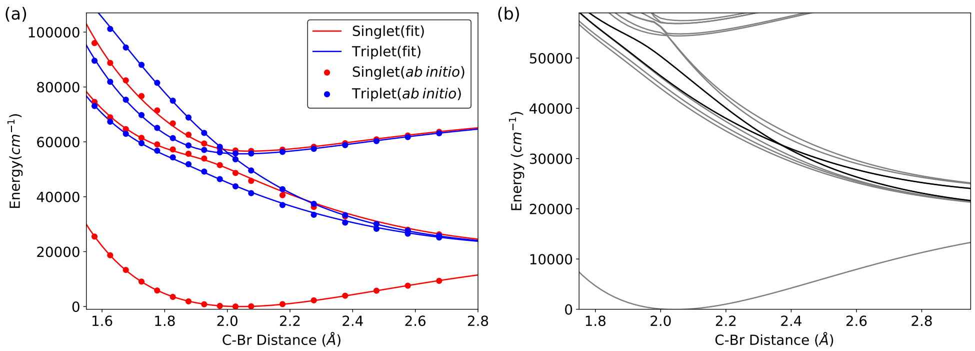

Supplementary Figure 5: Potential energy surfaces of CH_3Br along C-Br distance coordinate, with the remaining degrees-of-freedom frozen at the ground state equilibrium geometry. (a) Comparison between the *ab initio* spin-free adiabatic energies and the same values computed from the sub-block eigenvalues of the diabatic Hamiltonian. (b) Energy of spin-orbit states predicted by the diabatic Hamiltonian. The $^3Q_{0^+}$ and $^1Q_1$ states are highlighted.

Construction of Diabatic Hamiltonian

Analytic diabatic Hamiltonian matrices are constructed from *ab initio* energies and spin-orbit matrix elements calculated at SA-CASSCF level on a 1-D grid along the C-Br stretching coordinate. The CH_3 moiety is frozen at the equilibrium geometry of CH_3Br. Previous dynamics simulations on this system[6] ignored nonadiabatic couplings between spin-free adiabatic states. In this work, such interactions are addressed through a diabatization process. The diabatic states are constructed by diagonalizing the R^2 operator, centered at the carbon atom, among the excited states within each symmetry block[7]. This particular choice of diabatization method ensures that diabatic states maximally separate the large radius Rydberg states from the more compact valence excited states. An asymptotic ansatz [8] is adopted for the spin-orbit couplings between diabatic states, which is fit to the spin-orbit matrix elements between CASSCF states at the dissociation limit and the Frank-Condon region. The energies of spin-free singlet and triplet states and spin-orbit eigenstates predicted by the diabatic Hamiltonian are shown in Fig 5.

Wavepacket Dynamics

To obtain the excited state wavepacket dynamics, we solve the 1D time-dependent Schrödinger equation of the Hamiltonian

$$\hat{H} = \frac{1}{2\mu}\hat{\nabla}_R^2 \otimes \hat{\mathbf{1}}_d + \sum_{i,j} |i\rangle W_{i,j}(R) \langle j| \tag{2}$$

where μ is the reduced mass of CH_3Br associated with the C-Br stretching motion, R is the C-Br distance, W_{ij} contains the energies and off-diagonal matrix elements of the diabatic model described in the previous section, $\hat{\mathbf{1}}_d = \sum_i |i\rangle\langle i|$ is the unit operator in the discrete space of the diabatic electronic states, and $|i\rangle$ refers to the ith diabatic electronic state. The summations run over all 13 spin-orbit states including the valence states and the $5s$ Rydberg

states.

As opposed to previous studies on the photodissociation of CH_3Br[5, 6], the full information regarding the time evolution of the wave function is required in this work in order to generate the ATAS. Here, the nonadiabatic dynamics simulations are performed with Split-Operator Fourier Transform method[9] on a 1-d grid with 4096 grid points from 2.0 to 20 a.u., which corresponds to a tight spacing of 2.33×10^{-3}Å between grid points, using the analytic diabatic Hamiltonian. To prevent unphysical reflection at the grid boundary, Complex Absorbing Potentials(CAP) in the form of $-iU_0/\cosh^2 \alpha x$ are used[10]. The spatial parameter $\alpha = 0.5\text{Å}^{-1}$ limits the CAPs to regions near the asymptote, and the strength parameter $U_0 = 800\text{cm}^{-1}$ is the minimum value that is sufficient to prevent boundary artifacts. The magnitude of the wave function absorbed at the boundary also facilitates the computation of dissociation quantum yield and Br/Br* branching ratios. The ground vibrational wave packet is first prepared on the ground electronic state, then lifted to the target excited state. The wave packet is then propagated for 7000 a.u. (169 fs) using time steps of 2 a.u. (0.048 fs).

The dynamics simulations (Fig. 6) are performed with initial population on each adiabatic state as the initial condition. When Rydberg states are used as the initial state, the wave packets undergo nearly harmonic oscillations. The majority of the population is retained on the Rydberg state after the duration of the dynamic simulation, with a small population transferred to lower states. The Rydberg 5s state with $E(\Omega = 1)$ symmetry and $E_{1/2}$ cation core configuration has the highest dissociation yield of 0.077 for Br* and 0.016 for Br channel (Fig. 6). The other Rydberg state with significant transition dipole, with the same symmetry and $E_{3/2}$ core configuration, has a even lower total dissociation yield of 0.014 combining the two channels. Moreover, the dissociation process from Rydberg states is extended through a long period of time, as opposed to the dissociation process from valence excited states which occurs within a small time window. The transition dipole from Rydberg states to core-excited states is more than an order of magnitude lower than that from the valence excited states, and the resulting ATAS two orders of magnitude weaker. The transient signal from valence states during the dissociation process is also expected to be extremely weak, due to the extremely low probability density in the valence region at any given time. As a result, only very weak ATAS signal is obtained from these initial conditions.

Therefore we focus on the initial condition with valence excited states as the initial state. States with significant transition dipoles from the ground state include ${}^3Q_{0^+}$ state with parallel transition, and ${}^1Q_1$ and ${}^4E$ states with perpendicular transitions. With ${}^3Q_{0^+}$ as the initial condition, the dynamics of the adiabatic wave packets retains a Gaussian profile with gradual dispersion over time. A nearly Gaussian-shaped wave packet is created on the lower state near the conical intersection between ${}^3Q_{0^+}$ and ${}^1Q_1$ states and gaining most of the population between 10 and 20 fs, see Fig 6. The quantum yield in the Br* channel is 78.8% and the Br channel 20.7% after 160 fs simulation. In the parallel direction, ${}^1Q_1$ state has the largest transition dipole. The dissociation dynamics from this state is similarly ultrafast and nonadiabatic in nature, with the Br channel dominating with 76.5% yield and Br* channel 22.4%. With the ${}^3Q_{0^+}$ dynamics matching with experimentally observed branching ratio, significant initial population in ${}^1Q_1$ state can be ruled out.

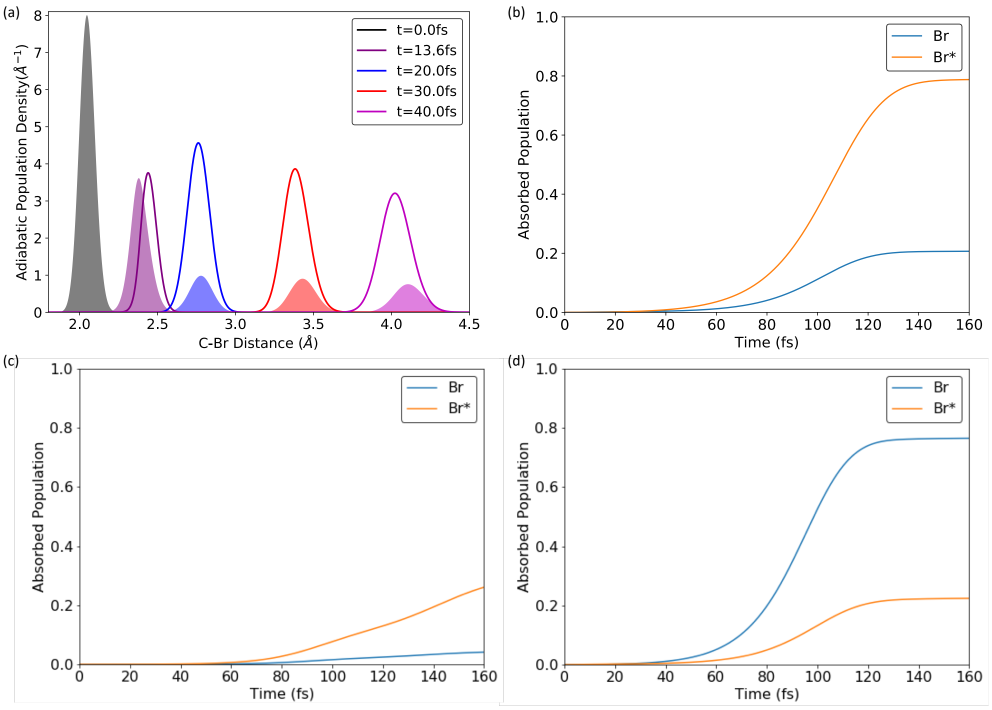

Supplementary Figure 6: 1D full quantum nonadiabatic dynamics of the photodissociation of CH_3Br starting from the $^3Q_{0+}$ state. (a) Snapshots of adiabatic population density at different simulation time. Solid: lower adiabatic PES, correlating with $^3Q_{0+}$ at FC region and $^1Q_1$ at the asymptote. Hollow: upper adiabatic PES. (b) Br/Br* yield calculated from the population at the grid boundary from each electronic states with $^3Q_{0+}$ state as the initial excitation. (c) Similar to (b), with the Rydberg 5s state with $E(\Omega = 1)$ symmetry and $E_{1/2}$ cation core configuration as the initial excitation. (d) Similar to (b), with $^1Q_1$ state as the initial condition.

Simulation of ATAS

Here we discuss the calculation of the Transient Absorption Spectrum (TAS) of a time dependent wave function, with the form of a Born-Huang expansion

$$|\Phi(r; R, t)\rangle = \sum_J \chi_J(R, t)|\Psi_J(r; R)\rangle \tag{3}$$

Where $|\Phi(r; R, t)\rangle$ is the time-dependent state to be measured, and J indexes valence electronic states of the molecule. Here, the XUV probe is assumed to be instantaneous, which is justified by the wide spectrum (>10 eV) of the probe pulse. Omitting the small coherence contribution between different adiabatic states, previous methods for computing TAS for single state wave functions[11] can be generalized for state defined by Eq (3), or

$$\sigma(\omega, \tau) = \frac{2\pi\omega\Gamma}{c} \sum_J \sum_\alpha \int \frac{|\chi_J(R, \tau)|^2 |\langle \Psi_J(r; R)|\hat{\mu}|\Psi_\alpha(r; R)\rangle|^2}{\frac{\Gamma^2}{4} + [E_\alpha(R) - E_J(R) - \omega]^2} dR. \tag{4}$$

The index α iterates through the first set of Br core-excited states. In the quantum

dynamics simulations, it is found that the wave function consists of one single Gaussian-like wave packet on each adiabatic state. This allows the TAS formula to be simplified to a form similar to those previously derived for Surface-Hopping trajectories[12], or

$$\sigma(\omega,\tau) \approx \frac{2\pi\omega\Gamma}{c}\sum_J\sum_\alpha \frac{p_J|\mu_{J,\alpha}(R_J)|^2}{\frac{\Gamma^2}{4}+[E_\alpha(R_J)-E_J(R_J)-\omega]^2}. \tag{5}$$

Here, p_J is the population on adiabatic state J, $\rho_J(\tau) \equiv \int |\chi_J(R,\tau)|^2 dR$, $\mu_{J,\alpha}$ is the transition dipole moment from state J to state α: $\mu_{J,\alpha}(R) \equiv \langle \Psi_J(r;R)|\hat{\mu}|\Psi_\alpha(r;R)\rangle$, and $R_J(\tau)$ is the nuclear position expectation value of the J state component of wave function Φ at time τ, or

$$R_J(\tau) \equiv \frac{\int R|\chi_J(R,\tau)|^2 dR}{\rho_J(\tau)}$$

Due to the finite pulse duration and the finite width of the Gaussian wave packets, the TAS defined in Eq(4) needs to be convoluted both in time and in energy. The width of such convolution is determined from the measured TAS.

To avoid the need to compute the TAS on the dense 1D grid, the transition dipole magnitudes $|\mu_{J,\alpha}(R)|^2$ and energy differences $E_\alpha(R) - E_J(R)$ are computed at a moderate number of R values and interpolated to yield analytical approximations. These quantities are calculated at Restricted-Active-Space Configuration Interaction(RASCI) level for various C-Br bond lengths, using the def2-SVP basis set along with the Stuttgart relativistic effective core potential for Br. The orbitals are obtained from SA-CASSCF calculations of valence and Rydberg states. Thereafter, RASCI is performed to obtain singlet and triplet wave functions of valence, Rydberg and core excited states, allowing full excitations within the valence orbitals, but restricting occupation of 3d orbitals to be 9 for core excited states and 10 for valence states.

Spin orbit couplings are computed from the Br effective core potential, and 32 spin orbit states are obtained, among which 20 are core excited states. The incorporation of spin-orbit couplings among valence and core excited states is crucial since both the intensities and energy differences between observed bands are dominated by the spin-orbit couplings. In the FC region, the spin-orbit matrix elements are significantly smaller than the differences between electronic energies, and spin-orbit states retain nearly pure singlet or triplet characters. Such weak interaction scheme is reflected by the Mulliken symbols which label these states as singlets and triplets. However, as we approaching the atomic limit, the spin-orbit couplings become the dominant contribution. This switch in interaction schemes, occurring near the region around 3 Å, causes a drastic change in the transition intensities. The Br $^2P_{1/2} \rightarrow {}^2D_{5/2}$ transition, which is forbidden at asymptotic limit, becomes allowed in FC region.

Supplementary References

[1] M. Mazzoni and M. Pettini, Phys. Lett. **85A**, 331 (1981).

[2] E. R. Hosler and S. R. Leone, Phys. Rev. A **88**, 023420 (2013).

[3] H.-J. Werner, P. J. Knowles, G. Knizia, F. R. Manby, M. Schütz, *et al.*, “Molpro, version 2015.1.0, a package of ab initio programs,” (2015), http://www.molpro.net.

[4] K. A. Peterson, D. Figgen, E. Goll, H. Stoll, and M. Dolg, “bibfield journal “bibinfo journal The Journal of Chemical Physics“ “textbf “bibinfo volume 119,“ “bibinfo pages 11113 (“bibinfo year 2003).

[5] C. Escure, T. Leininger, and B. Lepetit, “bibfield journal “bibinfo journal The Journal of Chemical Physics“ “textbf “bibinfo volume 130,“ “bibinfo pages 244306 (“bibinfo year 2009“natexlaba).

[6] C. Escure, T. Leininger, and B. Lepetit, The Journal of Chemical Physics **130**, 244305 (2009).

[7] D. R. Yarkony, “bibfield journal “bibinfo journal The Journal of Physical Chemistry A“ “textbf “bibinfo volume 102,“ “bibinfo pages 8073 (“bibinfo year 1998).

[8] H. Ndome, R. Welsch, and W. Eisfeld, J. Chem. Phys. **136**, 034103 (2012).

[9] M. Feit, J. Fleck, and A. Steiger, “bibfield journal “bibinfo journal Journal of Computational Physics“ “textbf “bibinfo volume 47,“ “bibinfo pages 412 (“bibinfo year 1982).

[10] R. Kosloff and D. Kosloff, Journal of Computational Physics **63**, 363 (1986).

[11] S.-Y. Lee, W. Pollard, and R. A. Mathies, Chem. Phys. Lett. **160**, 531 (1989).

[12] Z. Li, M. El-Amine Madjet, O. Vendrell, and R. Santra, Faraday Discuss. **171**, 457 (2014).
